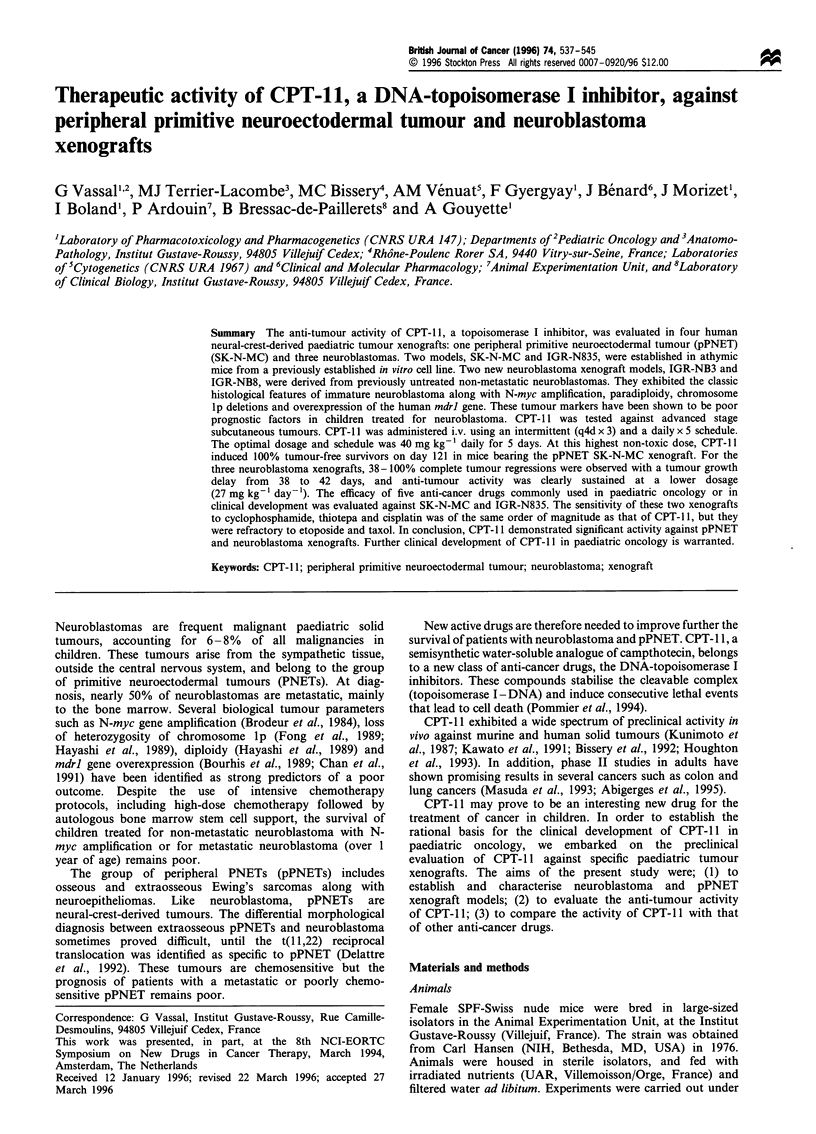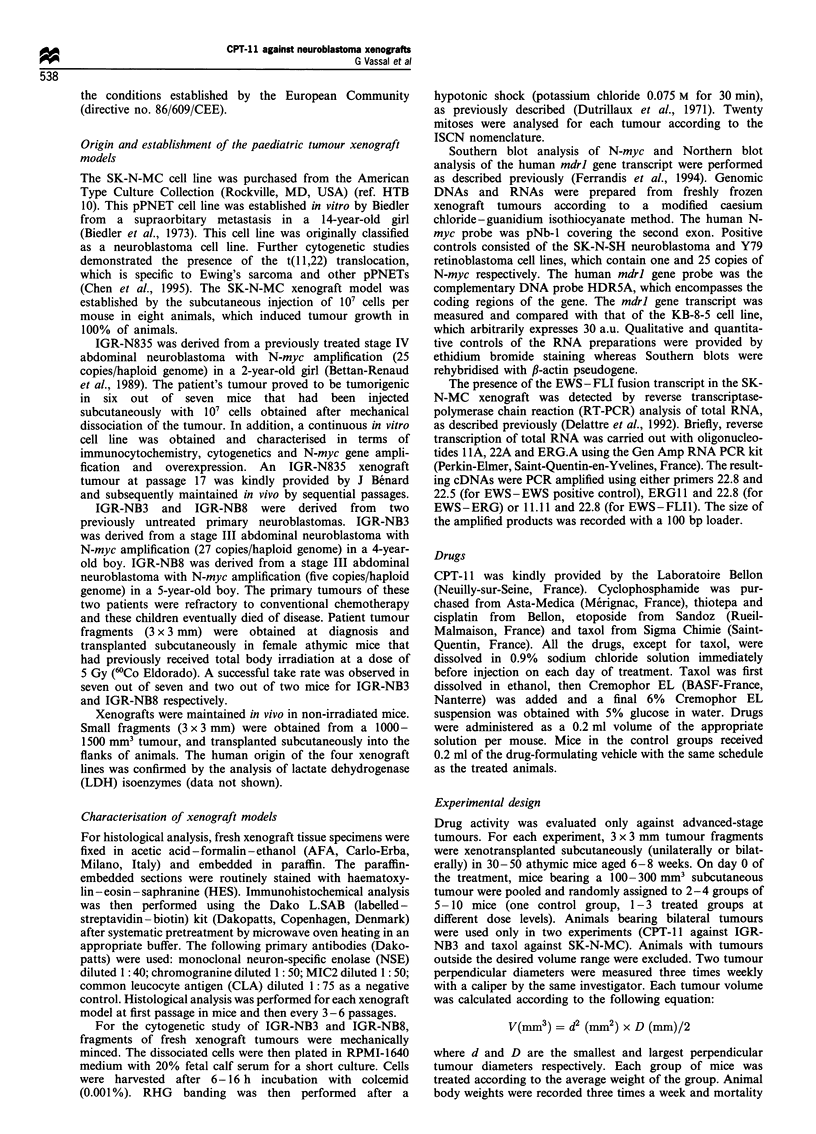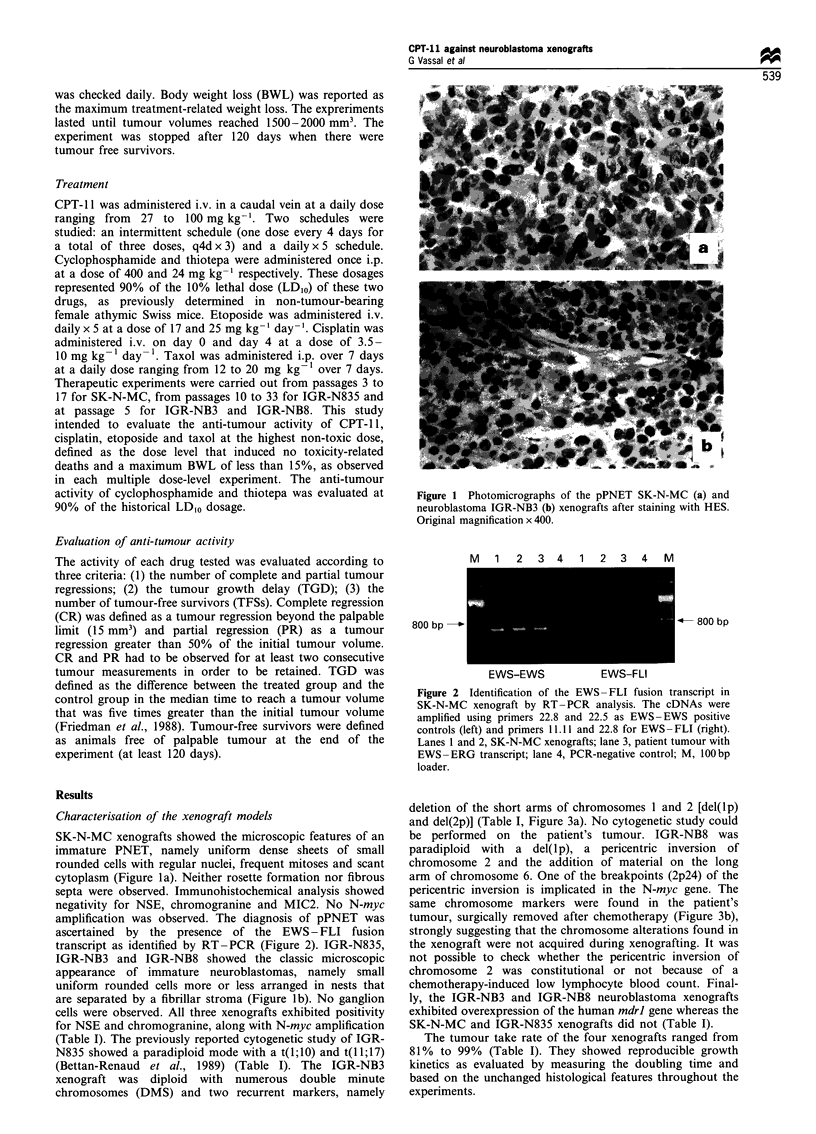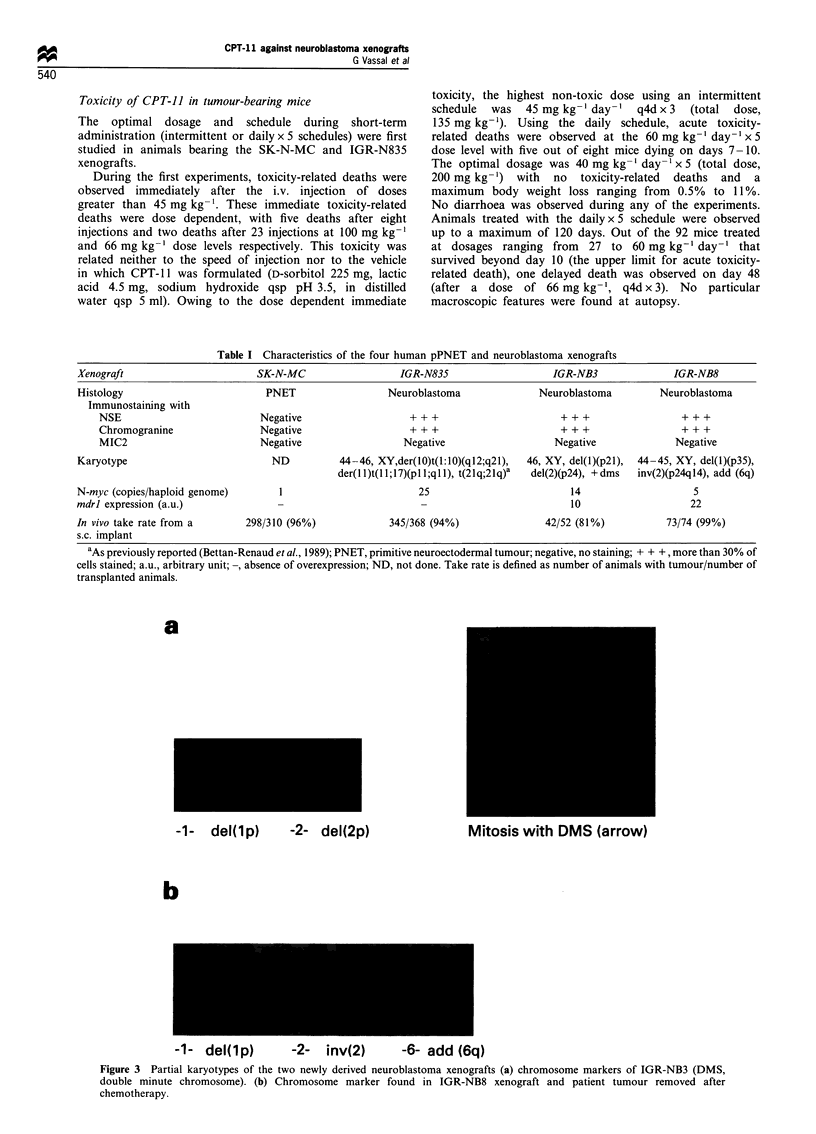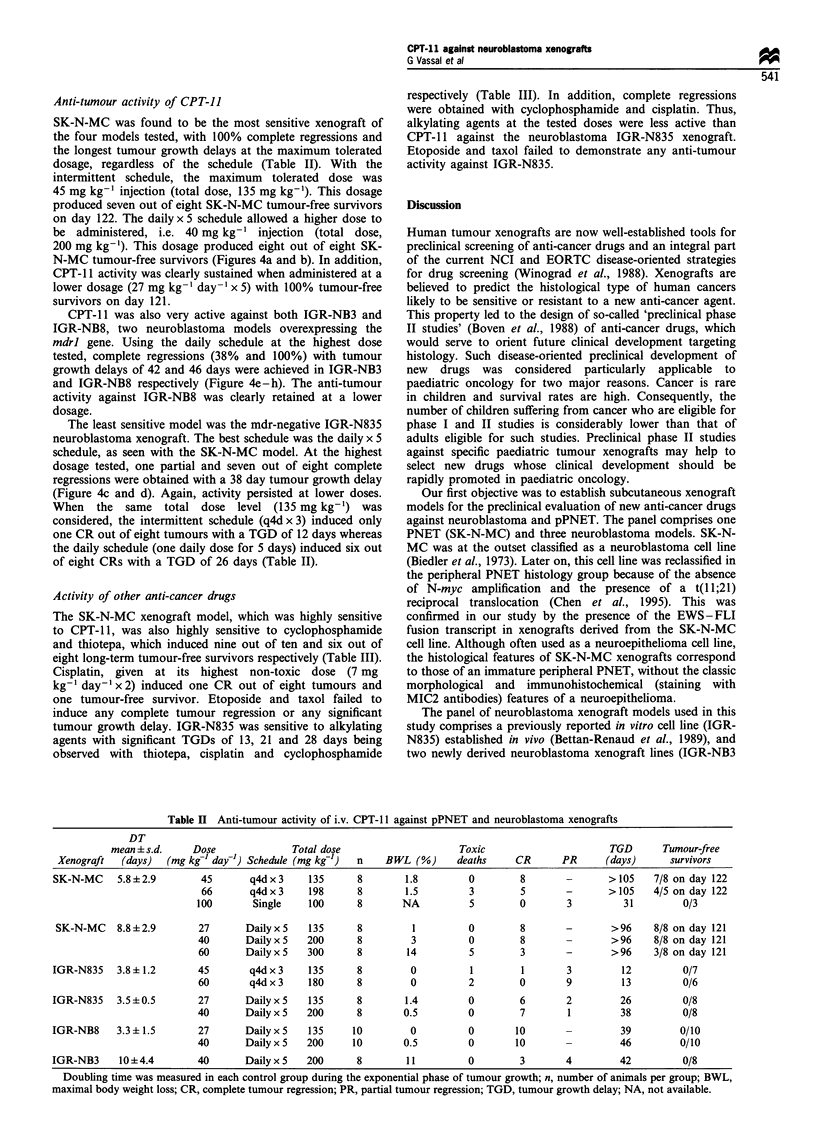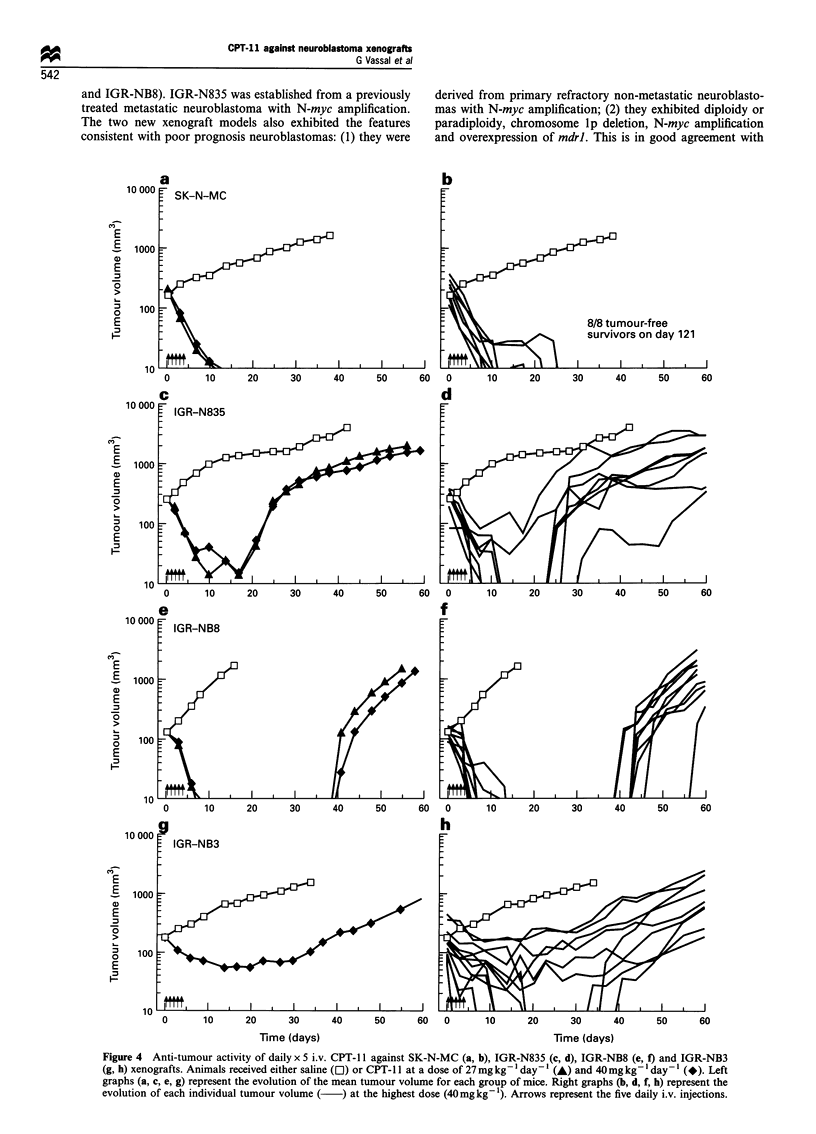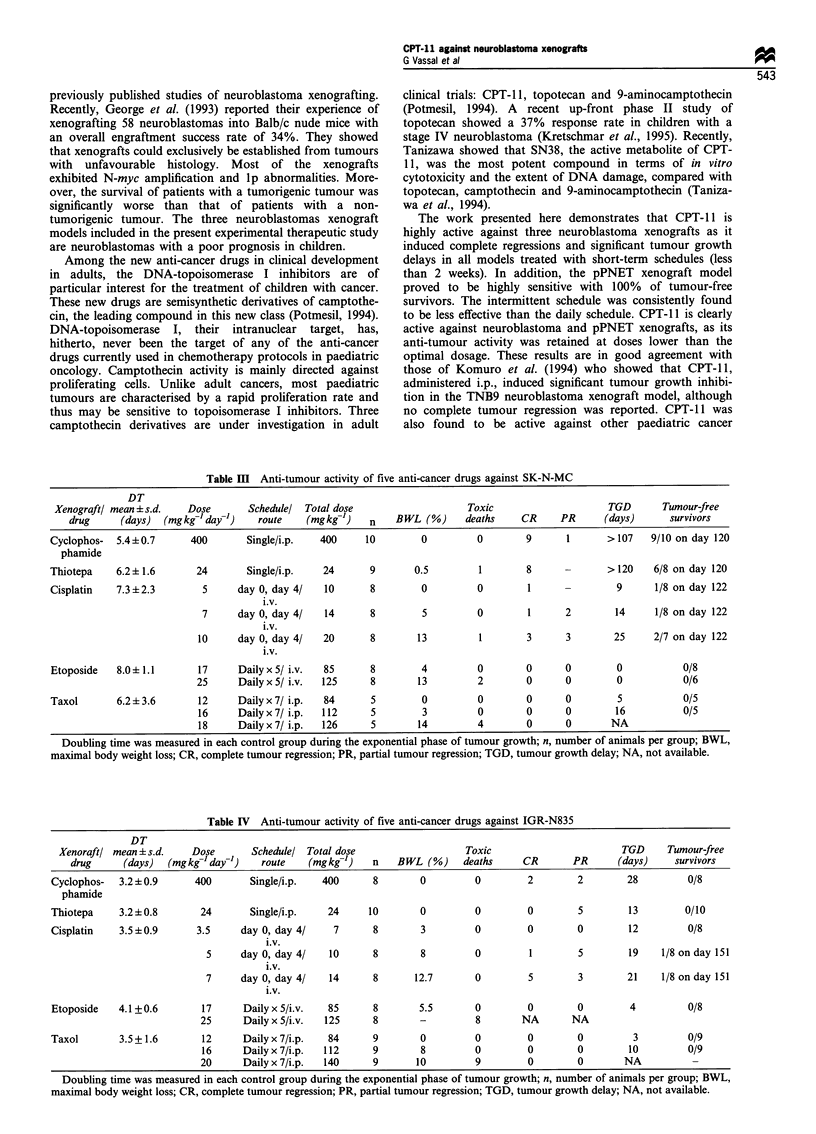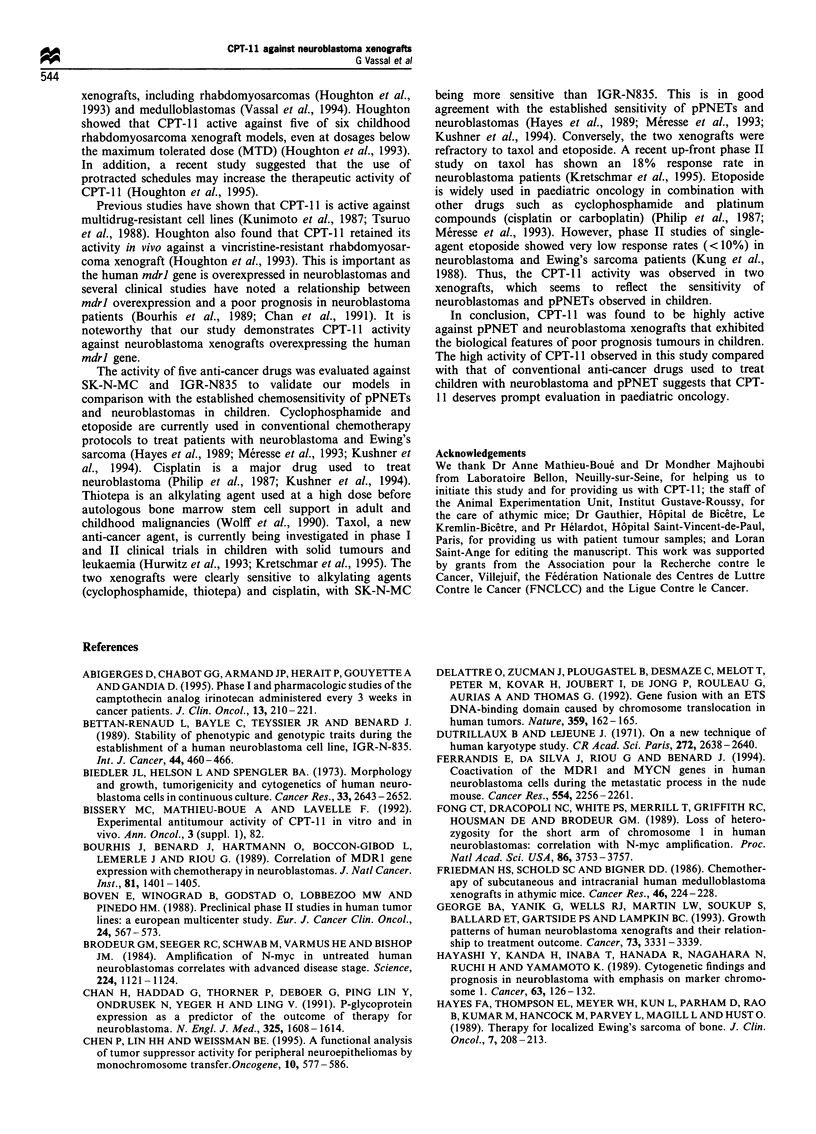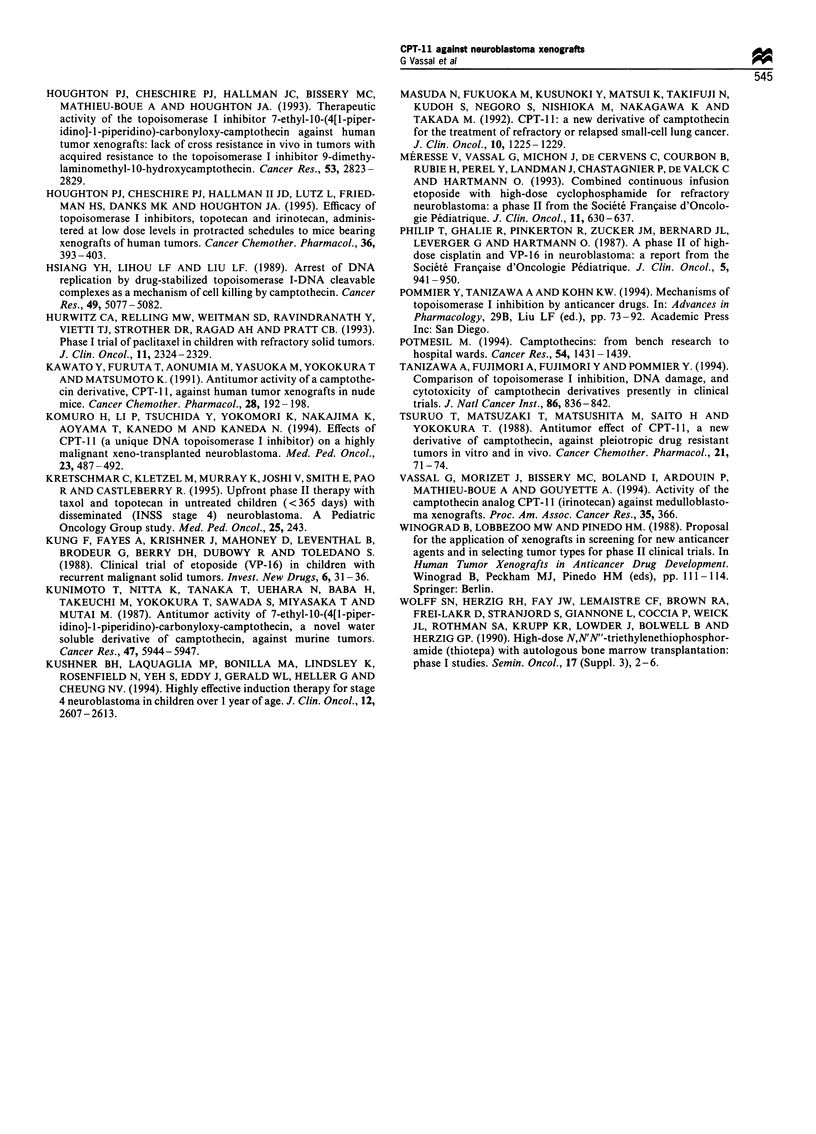# Therapeutic activity of CPT-11, a DNA-topoisomerase I inhibitor, against peripheral primitive neuroectodermal tumour and neuroblastoma xenografts.

**DOI:** 10.1038/bjc.1996.398

**Published:** 1996-08

**Authors:** G. Vassal, M. J. Terrier-Lacombe, M. C. Bissery, A. M. Vénuat, F. Gyergyay, J. Bénard, J. Morizet, I. Boland, P. Ardouin, B. Bressac-de-Paillerets, A. Gouyette

**Affiliations:** Department of Pediatric Oncology, Institut Gustave-Roussy, Villejuif, France.

## Abstract

**Images:**


British Journal of Cancer (1996) 74, 537-545
© 1996 Stockton Press All rights reserved 0007-0920/96 $12.00

### Therapeutic activity of CPT-11, a DNA-topoisomerase I inhibitor, against peripheral primitive neuroectodermal tumour and neuroblastoma xenografts

G Vassal[1,2], MJ Terrier-Lacombe[3], MC Bissery[4], AM Vénuat[5], F Gyergyay[1], J Bénard[6], J Morizet[1], I Boland[1], P Ardouin[7], B Bressac-de-Paillerets[8] and A Gouyette[1]

*[1]Laboratory of Pharmacotoxicology and Pharmacogenetics (CNRS URA 147); Departments of [2]Pediatric Oncology and [3]Anatomo-Pathology, Institut Gustave-Roussy, 94805 Villejuif Cedex; [4]Rhône-Poulenc Rorer SA, 9440 Vitry-sur-Seine, France; Laboratories of [5]Cytogenetics (CNRS URA 1967) and [6]Clinical and Molecular Pharmacology; [7]Animal Experimentation Unit, and [8]Laboratory of Clinical Biology, Institut Gustave-Roussy, 94805 Villejuif Cedex, France.*

**Summary** The anti-tumour activity of CPT-11, a topoisomerase I inhibitor, was evaluated in four human neural-crest-derived paediatric tumour xenografts: one peripheral primitive neuroectodermal tumour (pPNET) (SK-N-MC) and three neuroblastomas. Two models, SK-N-MC and IGR-N835, were established in athymic mice from a previously established *in vitro* cell line. Two new neuroblastoma xenograft models, IGR-NB3 and IGR-NB8, were derived from previously untreated non-metastatic neuroblastomas. They exhibited the classic histological features of immature neuroblastoma along with N-*myc* amplification, paradiploidy, chromosome 1p deletions and overexpression of the human *mdr1* gene. These tumour markers have been shown to be poor prognostic factors in children treated for neuroblastoma. CPT-11 was tested against advanced stage subcutaneous tumours. CPT-11 was administered i.v. using an intermittent (q4d × 3) and a daily × 5 schedule. The optimal dosage and schedule was 40 mg $kg^{-1}$ daily for 5 days. At this highest non-toxic dose, CPT-11 induced 100% tumour-free survivors on day 121 in mice bearing the pPNET SK-N-MC xenograft. For the three neuroblastoma xenografts, 38–100% complete tumour regressions were observed with a tumour growth delay from 38 to 42 days, and anti-tumour activity was clearly sustained at a lower dosage (27 mg $kg^{-1}$ $day^{-1}$). The efficacy of five anti-cancer drugs commonly used in paediatric oncology or in clinical development was evaluated against SK-N-MC and IGR-N835. The sensitivity of these two xenografts to cyclophosphamide, thiotepa and cisplatin was of the same order of magnitude as that of CPT-11, but they were refractory to etoposide and taxol. In conclusion, CPT-11 demonstrated significant activity against pPNET and neuroblastoma xenografts. Further clinical development of CPT-11 in paediatric oncology is warranted.

**Keywords:** CPT-11; peripheral primitive neuroectodermal tumour; neuroblastoma; xenograft

Neuroblastomas are frequent malignant paediatric solid tumours, accounting for 6–8% of all malignancies in children. These tumours arise from the sympathetic tissue, outside the central nervous system, and belong to the group of primitive neuroectodermal tumours (PNETs). At diagnosis, nearly 50% of neuroblastomas are metastatic, mainly to the bone marrow. Several biological tumour parameters such as N-*myc* gene amplification (Brodeur *et al.*, 1984), loss of heterozygosity of chromosome 1p (Fong *et al.*, 1989; Hayashi *et al.*, 1989), diploidy (Hayashi *et al.*, 1989) and *mdr1* gene overexpression (Bourhis *et al.*, 1989; Chan *et al.*, 1991) have been identified as strong predictors of a poor outcome. Despite the use of intensive chemotherapy protocols, including high-dose chemotherapy followed by autologous bone marrow stem cell support, the survival of children treated for non-metastatic neuroblastoma with N-*myc* amplification or for metastatic neuroblastoma (over 1 year of age) remains poor.

The group of peripheral PNETs (pPNETs) includes osseous and extraosseous Ewing's sarcomas along with neuroepitheliomas. Like neuroblastoma, pPNETs are neural-crest-derived tumours. The differential morphological diagnosis between extraosseous pPNETs and neuroblastoma sometimes proved difficult, until the t(11,22) reciprocal translocation was identified as specific to pPNET (Delattre *et al.*, 1992). These tumours are chemosensitive but the prognosis of patients with a metastatic or poorly chemosensitive pPNET remains poor.

Correspondence: G Vassal, Institut Gustave-Roussy, Rue Camille-Desmoulins, 94805 Villejuif Cedex, France
This work was presented, in part, at the 8th NCI-EORTC Symposium on New Drugs in Cancer Therapy, March 1994, Amsterdam, The Netherlands
Received 12 January 1996; revised 22 March 1996; accepted 27 March 1996

New active drugs are therefore needed to improve further the survival of patients with neuroblastoma and pPNET. CPT-11, a semisynthetic water-soluble analogue of campthotecin, belongs to a new class of anti-cancer drugs, the DNA-topoisomerase I inhibitors. These compounds stabilise the cleavable complex (topoisomerase I–DNA) and induce consecutive lethal events that lead to cell death (Pommier *et al.*, 1994).

CPT-11 exhibited a wide spectrum of preclinical activity *in vivo* against murine and human solid tumours (Kunimoto *et al.*, 1987; Kawato *et al.*, 1991; Bissery *et al.*, 1992; Houghton *et al.*, 1993). In addition, phase II studies in adults have shown promising results in several cancers such as colon and lung cancers (Masuda *et al.*, 1993; Abigerges *et al.*, 1995).

CPT-11 may prove to be an interesting new drug for the treatment of cancer in children. In order to establish the rational basis for the clinical development of CPT-11 in paediatric oncology, we embarked on the preclinical evaluation of CPT-11 against specific paediatric tumour xenografts. The aims of the present study were; (1) to establish and characterise neuroblastoma and pPNET xenograft models; (2) to evaluate the anti-tumour activity of CPT-11; (3) to compare the activity of CPT-11 with that of other anti-cancer drugs.

### Materials and methods

### *Animals*

Female SPF-Swiss nude mice were bred in large-sized isolators in the Animal Experimentation Unit, at the Institut Gustave-Roussy (Villejuif, France). The strain was obtained from Carl Hansen (NIH, Bethesda, MD, USA) in 1976. Animals were housed in sterile isolators, and fed with irradiated nutrients (UAR, Villemoisson/Orge, France) and filtered water *ad libitum*. Experiments were carried out under the conditions established by the European Community (directive no. 86/609/CEE).

*Origin and establishment of the paediatric tumour xenograft models*

The SK-N-MC cell line was purchased from the American Type Culture Collection (Rockville, MD, USA) (ref. HTB 10). This pPNET cell line was established *in vitro* by Biedler from a supraorbitary metastasis in a 14-year-old girl (Biedler *et al.*, 1973). This cell line was originally classified as a neuroblastoma cell line. Further cytogenetic studies demonstrated the presence of the t(11,22) translocation, which is specific to Ewing's sarcoma and other pPNETs (Chen *et al.*, 1995). The SK-N-MC xenograft model was established by the subcutaneous injection of $10^7$ cells per mouse in eight animals, which induced tumour growth in 100% of animals.

IGR-N835 was derived from a previously treated stage IV abdominal neuroblastoma with N-*myc* amplification (25 copies/haploid genome) in a 2-year-old girl (Bettan-Renaud *et al.*, 1989). The patient's tumour proved to be tumorigenic in six out of seven mice that had been injected subcutaneously with $10^7$ cells obtained after mechanical dissociation of the tumour. In addition, a continuous *in vitro* cell line was obtained and characterised in terms of immunocytochemistry, cytogenetics and N-*myc* gene amplification and overexpression. An IGR-N835 xenograft tumour at passage 17 was kindly provided by J Bénard and subsequently maintained *in vivo* by sequential passages.

IGR-NB3 and IGR-NB8 were derived from two previously untreated primary neuroblastomas. IGR-NB3 was derived from a stage III abdominal neuroblastoma with N-*myc* amplification (27 copies/haploid genome) in a 4-year-old boy. IGR-NB8 was derived from a stage III abdominal neuroblastoma with N-*myc* amplification (five copies/haploid genome) in a 5-year-old boy. The primary tumours of these two patients were refractory to conventional chemotherapy and these children eventually died of disease. Patient tumour fragments (3 x 3 mm) were obtained at diagnosis and transplanted subcutaneously in female athymic mice that had previously received total body irradiation at a dose of 5 Gy ($^{60}$Co Eldorado). A successful take rate was observed in seven out of seven and two out of two mice for IGR-NB3 and IGR-NB8 respectively.

Xenografts were maintained *in vivo* in non-irradiated mice. Small fragments (3 x 3 mm) were obtained from a 1000–1500 $mm^3$ tumour, and transplanted subcutaneously into the flanks of animals. The human origin of the four xenograft lines was confirmed by the analysis of lactate dehydrogenase (LDH) isoenzymes (data not shown).

*Characterisation of xenograft models*

For histological analysis, fresh xenograft tissue specimens were fixed in acetic acid–formalin–ethanol (AFA, Carlo-Erba, Milano, Italy) and embedded in paraffin. The paraffin-embedded sections were routinely stained with haematoxylin–eosin–saphranine (HES). Immunohistochemical analysis was then performed using the Dako L.SAB (labelled–streptavidin–biotin) kit (Dakopatts, Copenhagen, Denmark) after systematic pretreatment by microwave oven heating in an appropriate buffer. The following primary antibodies (Dakopatts) were used: monoclonal neuron-specific enolase (NSE) diluted 1 : 40; chromogranine diluted 1 : 50; MIC2 diluted 1 : 50; common leucocyte antigen (CLA) diluted 1 : 75 as a negative control. Histological analysis was performed for each xenograft model at first passage in mice and then every 3–6 passages.

For the cytogenetic study of IGR-NB3 and IGR-NB8, fragments of fresh xenograft tumours were mechanically minced. The dissociated cells were then plated in RPMI-1640 medium with 20% fetal calf serum for a short culture. Cells were harvested after 6–16 h incubation with colcemid (0.001%). RHG banding was then performed after a hypotonic shock (potassium chloride 0.075 M for 30 min), as previously described (Dutrillaux *et al.*, 1971). Twenty mitoses were analysed for each tumour according to the ISCN nomenclature.

Southern blot analysis of N-*myc* and Northern blot analysis of the human *mdr1* gene transcript were performed as described previously (Ferrandis *et al.*, 1994). Genomic DNAs and RNAs were prepared from freshly frozen xenograft tumours according to a modified caesium chloride–guanidium isothiocyanate method. The human N-*myc* probe was pNb-1 covering the second exon. Positive controls consisted of the SK-N-SH neuroblastoma and Y79 retinoblastoma cell lines, which contain one and 25 copies of N-*myc* respectively. The human *mdr1* gene probe was the complementary DNA probe HDR5A, which encompasses the coding regions of the gene. The *mdr1* gene transcript was measured and compared with that of the KB-8-5 cell line, which arbitrarily expresses 30 a.u. Qualitative and quantitative controls of the RNA preparations were provided by ethidium bromide staining whereas Southern blots were rehybridised with $\beta$-actin pseudogene.

The presence of the EWS–FLI fusion transcript in the SK-N-MC xenograft was detected by reverse transcriptase-polymerase chain reaction (RT-PCR) analysis of total RNA, as described previously (Delattre *et al.*, 1992). Briefly, reverse transcription of total RNA was carried out with oligonucleotides 11A, 22A and ERG.A using the Gen Amp RNA PCR kit (Perkin-Elmer, Saint-Quentin-en-Yvelines, France). The resulting cDNAs were PCR amplified using either primers 22.8 and 22.5 (for EWS–EWS positive control), ERG11 and 22.8 (for EWS–ERG) or 11.11 and 22.8 (for EWS–FLI1). The size of the amplified products was recorded with a 100 bp loader.

*Drugs*

CPT-11 was kindly provided by the Laboratoire Bellon (Neuilly-sur-Seine, France). Cyclophosphamide was purchased from Asta-Medica (Mérignac, France), thiotepa and cisplatin from Bellon, etoposide from Sandoz (Rueil-Malmaison, France) and taxol from Sigma Chimie (Saint-Quentin, France). All the drugs, except for taxol, were dissolved in 0.9% sodium chloride solution immediately before injection on each day of treatment. Taxol was first dissolved in ethanol, then Cremophor EL (BASF-France, Nanterre) was added and a final 6% Cremophor EL suspension was obtained with 5% glucose in water. Drugs were administered as a 0.2 ml volume of the appropriate solution per mouse. Mice in the control groups received 0.2 ml of the drug-formulating vehicle with the same schedule as the treated animals.

*Experimental design*

Drug activity was evaluated only against advanced-stage tumours. For each experiment, 3 x 3 mm tumour fragments were xenotransplanted subcutaneously (unilaterally or bilaterally) in 30–50 athymic mice aged 6–8 weeks. On day 0 of the treatment, mice bearing a 100–300 $mm^3$ subcutaneous tumour were pooled and randomly assigned to 2–4 groups of 5–10 mice (one control group, 1–3 treated groups at different dose levels). Animals bearing bilateral tumours were used only in two experiments (CPT-11 against IGR-NB3 and taxol against SK-N-MC). Animals with tumours outside the desired volume range were excluded. Two tumour perpendicular diameters were measured three times weekly with a caliper by the same investigator. Each tumour volume was calculated according to the following equation:

$$V(\text{mm}^3) = d^2\ (\text{mm}^2) \times D\ (\text{mm})/2$$

where $d$ and $D$ are the smallest and largest perpendicular tumour diameters respectively. Each group of mice was treated according to the average weight of the group. Animal body weights were recorded three times a week and mortality was checked daily. Body weight loss (BWL) was reported as the maximum treatment-related weight loss. The expreriments lasted until tumour volumes reached 1500–2000 mm$^3$. The experiment was stopped after 120 days when there were tumour free survivors.

*Treatment*

CPT-11 was administered i.v. in a caudal vein at a daily dose ranging from 27 to 100 mg kg$^{-1}$. Two schedules were studied: an intermittent schedule (one dose every 4 days for a total of three doses, q4d × 3) and a daily × 5 schedule. Cyclophosphamide and thiotepa were administered once i.p. at a dose of 400 and 24 mg kg$^{-1}$ respectively. These dosages represented 90% of the 10% lethal dose ($LD_{10}$) of these two drugs, as previously determined in non-tumour-bearing female athymic Swiss mice. Etoposide was administered i.v. daily × 5 at a dose of 17 and 25 mg kg$^{-1}$ day$^{-1}$. Cisplatin was administered i.v. on day 0 and day 4 at a dose of 3.5–10 mg kg$^{-1}$ day$^{-1}$. Taxol was administered i.p. over 7 days at a daily dose ranging from 12 to 20 mg kg$^{-1}$ over 7 days. Therapeutic experiments were carried out from passages 3 to 17 for SK-N-MC, from passages 10 to 33 for IGR-N835 and at passage 5 for IGR-NB3 and IGR-NB8. This study intended to evaluate the anti-tumour activity of CPT-11, cisplatin, etoposide and taxol at the highest non-toxic dose, defined as the dose level that induced no toxicity-related deaths and a maximum BWL of less than 15%, as observed in each multiple dose-level experiment. The anti-tumour activity of cyclophosphamide and thiotepa was evaluated at 90% of the historical $LD_{10}$ dosage.

*Evaluation of anti-tumour activity*

The activity of each drug tested was evaluated according to three criteria: (1) the number of complete and partial tumour regressions; (2) the tumour growth delay (TGD); (3) the number of tumour-free survivors (TFSs). Complete regression (CR) was defined as a tumour regression beyond the palpable limit (15 mm$^3$) and partial regression (PR) as a tumour regression greater than 50% of the initial tumour volume. CR and PR had to be observed for at least two consecutive tumour measurements in order to be retained. TGD was defined as the difference between the treated group and the control group in the median time to reach a tumour volume that was five times greater than the initial tumour volume (Friedman *et al.*, 1988). Tumour-free survivors were defined as animals free of palpable tumour at the end of the experiment (at least 120 days).

**Figure 1** Photomicrographs of the pPNET SK-N-MC (**a**) and neuroblastoma IGR-NB3 (**b**) xenografts after staining with HES. Original magnification × 400.

**Figure 2** Identification of the EWS–FLI fusion transcript in SK-N-MC xenograft by RT–PCR analysis. The cDNAs were amplified using primers 22.8 and 22.5 as EWS–EWS positive controls (left) and primers 11.11 and 22.8 for EWS–FLI (right). Lanes 1 and 2, SK-N-MC xenografts; lane 3, patient tumour with EWS–ERG transcript; lane 4, PCR-negative control; M, 100 bp loader.

### Results

*Characterisation of the xenograft models*

SK-N-MC xenografts showed the microscopic features of an immature PNET, namely uniform dense sheets of small rounded cells with regular nuclei, frequent mitoses and scant cytoplasm (Figure 1a). Neither rosette formation nor fibrous septa were observed. Immunohistochemical analysis showed negativity for NSE, chromogranine and MIC2. No N-*myc* amplification was observed. The diagnosis of pPNET was ascertained by the presence of the EWS–FLI fusion transcript as identified by RT–PCR (Figure 2). IGR-N835, IGR-NB3 and IGR-NB8 showed the classic microscopic appearance of immature neuroblastomas, namely small uniform rounded cells more or less arranged in nests that are separated by a fibrillar stroma (Figure 1b). No ganglion cells were observed. All three xenografts exhibited positivity for NSE and chromogranine, along with N-*myc* amplification (Table I). The previously reported cytogenetic study of IGR-N835 showed a paradiploid mode with a t(1;10) and t(11;17) (Bettan-Renaud *et al.*, 1989) (Table I). The IGR-NB3 xenograft was diploid with numerous double minute chromosomes (DMS) and two recurrent markers, namely deletion of the short arms of chromosomes 1 and 2 [del(1p) and del(2p)] (Table I, Figure 3a). No cytogenetic study could be performed on the patient's tumour. IGR-NB8 was paradiploid with a del(1p), a pericentric inversion of chromosome 2 and the addition of material on the long arm of chromosome 6. One of the breakpoints (2p24) of the pericentric inversion is implicated in the N-*myc* gene. The same chromosome markers were found in the patient's tumour, surgically removed after chemotherapy (Figure 3b), strongly suggesting that the chromosome alterations found in the xenograft were not acquired during xenografting. It was not possible to check whether the pericentric inversion of chromosome 2 was constitutional or not because of a chemotherapy-induced low lymphocyte blood count. Finally, the IGR-NB3 and IGR-NB8 neuroblastoma xenografts exhibited overexpression of the human *mdr1* gene whereas the SK-N-MC and IGR-N835 xenografts did not (Table I).

The tumour take rate of the four xenografts ranged from 81% to 99% (Table I). They showed reproducible growth kinetics as evaluated by measuring the doubling time and based on the unchanged histological features throughout the experiments.

*Toxicity of CPT-11 in tumour-bearing mice*

The optimal dosage and schedule during short-term administration (intermittent or daily × 5 schedules) were first studied in animals bearing the SK-N-MC and IGR-N835 xenografts.

During the first experiments, toxicity-related deaths were observed immediately after the i.v. injection of doses greater than 45 mg $kg^{-1}$. These immediate toxicity-related deaths were dose dependent, with five deaths after eight injections and two deaths after 23 injections at 100 mg $kg^{-1}$ and 66 mg $kg^{-1}$ dose levels respectively. This toxicity was related neither to the speed of injection nor to the vehicle in which CPT-11 was formulated (D-sorbitol 225 mg, lactic acid 4.5 mg, sodium hydroxide qsp pH 3.5, in distilled water qsp 5 ml). Owing to the dose dependent immediate toxicity, the highest non-toxic dose using an intermittent schedule was 45 mg $kg^{-1}$ $day^{-1}$ q4d × 3 (total dose, 135 mg $kg^{-1}$). Using the daily schedule, acute toxicity-related deaths were observed at the 60 mg $kg^{-1}$ $day^{-1}$ × 5 dose level with five out of eight mice dying on days 7–10. The optimal dosage was 40 mg $kg^{-1}$ $day^{-1}$ × 5 (total dose, 200 mg $kg^{-1}$) with no toxicity-related deaths and a maximum body weight loss ranging from 0.5% to 11%. No diarrhoea was observed during any of the experiments. Animals treated with the daily × 5 schedule were observed up to a maximum of 120 days. Out of the 92 mice treated at dosages ranging from 27 to 60 mg $kg^{-1}$ $day^{-1}$ that survived beyond day 10 (the upper limit for acute toxicity-related death), one delayed death was observed on day 48 (after a dose of 66 mg $kg^{-1}$, q4d × 3). No particular macroscopic features were found at autopsy.

**Table I** Characteristics of the four human pPNET and neuroblastoma xenografts

| *Xenograft* | *SK-N-MC* | *IGR-N835* | *IGR-NB3* | *IGR-NB8* |
|---|---|---|---|---|
| Histology | PNET | Neuroblastoma | Neuroblastoma | Neuroblastoma |
| Immunostaining with | | | | |
| NSE | Negative | + + + | + + + | + + + |
| Chromogranine | Negative | + + + | + + + | + + + |
| MIC2 | Negative | Negative | Negative | Negative |
| Karyotype | ND | 44–46, XY,der(10)t(1:10)(q12;q21), der(11)t(11;17)(p11;q11), t(21q;21q)[a] | 46, XY, del(1)(p21), del(2)(p24), +dms | 44–45, XY, del(1)(p35), inv(2)(p24q14), add (6q) |
| N-*myc* (copies/haploid genome) | 1 | 25 | 14 | 5 |
| *mdr1* expression (a.u.) | – | – | 10 | 22 |
| *In vivo* take rate from a s.c. implant | 298/310 (96%) | 345/368 (94%) | 42/52 (81%) | 73/74 (99%) |

[a]As previously reported (Bettan-Renaud *et al*., 1989); PNET, primitive neuroectodermal tumour; negative, no staining; + + +, more than 30% of cells stained; a.u., arbitrary unit; –, absence of overexpression; ND, not done. Take rate is defined as number of animals with tumour/number of transplanted animals.

**Figure 3** Partial karyotypes of the two newly derived neuroblastoma xenografts (**a**) chromosome markers of IGR-NB3 (DMS, double minute chromosome). (**b**) Chromosome marker found in IGR-NB8 xenograft and patient tumour removed after chemotherapy.

*Anti-tumour activity of CPT-11*

SK-N-MC was found to be the most sensitive xenograft of the four models tested, with 100% complete regressions and the longest tumour growth delays at the maximum tolerated dosage, regardless of the schedule (Table II). With the intermittent schedule, the maximum tolerated dose was 45 mg $kg^{-1}$ injection (total dose, 135 mg $kg^{-1}$). This dosage produced seven out of eight SK-N-MC tumour-free survivors on day 122. The daily × 5 schedule allowed a higher dose to be administered, i.e. 40 mg $kg^{-1}$ injection (total dose, 200 mg $kg^{-1}$). This dosage produced eight out of eight SK-N-MC tumour-free survivors (Figures 4a and b). In addition, CPT-11 activity was clearly sustained when administered at a lower dosage (27 mg $kg^{-1}$ $day^{-1}$ × 5) with 100% tumour-free survivors on day 121.

CPT-11 was also very active against both IGR-NB3 and IGR-NB8, two neuroblastoma models overexpressing the *mdr1* gene. Using the daily schedule at the highest dose tested, complete regressions (38% and 100%) with tumour growth delays of 42 and 46 days were achieved in IGR-NB3 and IGR-NB8 respectively (Figure 4e–h). The anti-tumour activity against IGR-NB8 was clearly retained at a lower dosage.

The least sensitive model was the mdr-negative IGR-N835 neuroblastoma xenograft. The best schedule was the daily × 5 schedule, as seen with the SK-N-MC model. At the highest dosage tested, one partial and seven out of eight complete regressions were obtained with a 38 day tumour growth delay (Figure 4c and d). Again, activity persisted at lower doses. When the same total dose level (135 mg $kg^{-1}$) was considered, the intermittent schedule (q4d × 3) induced only one CR out of eight tumours with a TGD of 12 days whereas the daily schedule (one daily dose for 5 days) induced six out of eight CRs with a TGD of 26 days (Table II).

*Activity of other anti-cancer drugs*

The SK-N-MC xenograft model, which was highly sensitive to CPT-11, was also highly sensitive to cyclophosphamide and thiotepa, which induced nine out of ten and six out of eight long-term tumour-free survivors respectively (Table III). Cisplatin, given at its highest non-toxic dose (7 mg $kg^{-1}$ $day^{-1}$ × 2) induced one CR out of eight tumours and one tumour-free survivor. Etoposide and taxol failed to induce any complete tumour regression or any significant tumour growth delay. IGR-N835 was sensitive to alkylating agents with significant TGDs of 13, 21 and 28 days being observed with thiotepa, cisplatin and cyclophosphamide respectively (Table III). In addition, complete regressions were obtained with cyclophosphamide and cisplatin. Thus, alkylating agents at the tested doses were less active than CPT-11 against the neuroblastoma IGR-N835 xenograft. Etoposide and taxol failed to demonstrate any anti-tumour activity against IGR-N835.

### Discussion

Human tumour xenografts are now well-established tools for preclinical screening of anti-cancer drugs and an integral part of the current NCI and EORTC disease-oriented strategies for drug screening (Winograd *et al.*, 1988). Xenografts are believed to predict the histological type of human cancers likely to be sensitive or resistant to a new anti-cancer agent. This property led to the design of so-called 'preclinical phase II studies' (Boven *et al.*, 1988) of anti-cancer drugs, which would serve to orient future clinical development targeting histology. Such disease-oriented preclinical development of new drugs was considered particularly applicable to paediatric oncology for two major reasons. Cancer is rare in children and survival rates are high. Consequently, the number of children suffering from cancer who are eligible for phase I and II studies is considerably lower than that of adults eligible for such studies. Preclinical phase II studies against specific paediatric tumour xenografts may help to select new drugs whose clinical development should be rapidly promoted in paediatric oncology.

Our first objective was to establish subcutaneous xenograft models for the preclinical evaluation of new anti-cancer drugs against neuroblastoma and pPNET. The panel comprises one PNET (SK-N-MC) and three neuroblastoma models. SK-N-MC was at the outset classified as a neuroblastoma cell line (Biedler *et al.*, 1973). Later on, this cell line was reclassified in the peripheral PNET histology group because of the absence of N-*myc* amplification and the presence of a t(11;21) reciprocal translocation (Chen *et al.*, 1995). This was confirmed in our study by the presence of the EWS–FLI fusion transcript in xenografts derived from the SK-N-MC cell line. Although often used as a neuroepithelioma cell line, the histological features of SK-N-MC xenografts correspond to those of an immature peripheral PNET, without the classic morphological and immunohistochemical (staining with MIC2 antibodies) features of a neuroepithelioma.

The panel of neuroblastoma xenograft models used in this study comprises a previously reported *in vitro* cell line (IGR-N835) established *in vivo* (Bettan-Renaud *et al.*, 1989), and two newly derived neuroblastoma xenograft lines (IGR-NB3

**Table II** Anti-tumour activity of i.v. CPT-11 against pPNET and neuroblastoma xenografts

| *Xenograft* | *DT mean ± s.d. (days)* | *Dose (mg $kg^{-1}$ $day^{-1}$)* | *Schedule* | *Total dose (mg $kg^{-1}$)* | n | *BWL (%)* | *Toxic deaths* | *CR* | *PR* | *TGD (days)* | *Tumour-free survivors* |
|---|---|---|---|---|---|---|---|---|---|---|---|
| SK-N-MC | 5.8 ± 2.9 | 45 | q4d × 3 | 135 | 8 | 1.8 | 0 | 8 | – | > 105 | 7/8 on day 122 |
| | | 66 | q4d × 3 | 198 | 8 | 1.5 | 3 | 5 | – | > 105 | 4/5 on day 122 |
| | | 100 | Single | 100 | 8 | NA | 5 | 0 | 3 | 31 | 0/3 |
| SK-N-MC | 8.8 ± 2.9 | 27 | Daily × 5 | 135 | 8 | 1 | 0 | 8 | – | >96 | 8/8 on day 121 |
| | | 40 | Daily × 5 | 200 | 8 | 3 | 0 | 8 | – | >96 | 8/8 on day 121 |
| | | 60 | Daily × 5 | 300 | 8 | 14 | 5 | 3 | – | >96 | 3/8 on day 121 |
| IGR-N835 | 3.8 ± 1.2 | 45 | q4d × 3 | 135 | 8 | 0 | 1 | 1 | 3 | 12 | 0/7 |
| | | 60 | q4d × 3 | 180 | 8 | 0 | 2 | 0 | 9 | 13 | 0/6 |
| IGR-N835 | 3.5 ± 0.5 | 27 | Daily × 5 | 135 | 8 | 1.4 | 0 | 6 | 2 | 26 | 0/8 |
| | | 40 | Daily × 5 | 200 | 8 | 0.5 | 0 | 7 | 1 | 38 | 0/8 |
| IGR-NB8 | 3.3 ± 1.5 | 27 | Daily × 5 | 135 | 10 | 0 | 0 | 10 | – | 39 | 0/10 |
| | | 40 | Daily × 5 | 200 | 10 | 0.5 | 0 | 10 | – | 46 | 0/10 |
| IGR-NB3 | 10 ± 4.4 | 40 | Daily × 5 | 200 | 8 | 11 | 0 | 3 | 4 | 42 | 0/8 |

Doubling time was measured in each control group during the exponential phase of tumour growth; *n*, number of animals per group; BWL, maximal body weight loss; CR, complete tumour regression; PR, partial tumour regression; TGD, tumour growth delay; NA, not available.

and IGR-NB8). IGR-N835 was established from a previously treated metastatic neuroblastoma with N-*myc* amplification. The two new xenograft models also exhibited the features consistent with poor prognosis neuroblastomas: (1) they were derived from primary refractory non-metastatic neuroblastomas with N-*myc* amplification; (2) they exhibited diploidy or paradiploidy, chromosome 1p deletion, N-*myc* amplification and overexpression of *mdr1*. This is in good agreement with

**Figure 4** Anti-tumour activity of daily × 5 i.v. CPT-11 against SK-N-MC (**a**, **b**), IGR-N835 (**c**, **d**), IGR-NB8 (**e**, **f**) and IGR-NB3 (**g**, **h**) xenografts. Animals received either saline (□) or CPT-11 at a dose of 27 mg kg$^{-1}$ day$^{-1}$ (▲) and 40 mg kg$^{-1}$ day$^{-1}$ (◆). Left graphs (**a**, **c**, **e**, **g**) represent the evolution of the mean tumour volume for each group of mice. Right graphs (**b**, **d**, **f**, **h**) represent the evolution of each individual tumour volume (——) at the highest dose (40 mg kg$^{-1}$). Arrows represent the five daily i.v. injections.

previously published studies of neuroblastoma xenografting. Recently, George *et al.* (1993) reported their experience of xenografting 58 neuroblastomas into Balb/c nude mice with an overall engraftment success rate of 34%. They showed that xenografts could exclusively be established from tumours with unfavourable histology. Most of the xenografts exhibited N-*myc* amplification and 1p abnormalities. Moreover, the survival of patients with a tumorigenic tumour was significantly worse than that of patients with a non-tumorigenic tumour. The three neuroblastomas xenograft models included in the present experimental therapeutic study are neuroblastomas with a poor prognosis in children.

Among the new anti-cancer drugs in clinical development in adults, the DNA-topoisomerase I inhibitors are of particular interest for the treatment of children with cancer. These new drugs are semisynthetic derivatives of camptothecin, the leading compound in this new class (Potmesil, 1994). DNA-topoisomerase I, their intranuclear target, has, hitherto, never been the target of any of the anti-cancer drugs currently used in chemotherapy protocols in paediatric oncology. Camptothecin activity is mainly directed against proliferating cells. Unlike adult cancers, most paediatric tumours are characterised by a rapid proliferation rate and thus may be sensitive to topoisomerase I inhibitors. Three camptothecin derivatives are under investigation in adult clinical trials: CPT-11, topotecan and 9-aminocamptothecin (Potmesil, 1994). A recent up-front phase II study of topotecan showed a 37% response rate in children with a stage IV neuroblastoma (Kretschmar *et al.*, 1995). Recently, Tanizawa showed that SN38, the active metabolite of CPT-11, was the most potent compound in terms of *in vitro* cytotoxicity and the extent of DNA damage, compared with topotecan, camptothecin and 9-aminocamptothecin (Tanizawa *et al.*, 1994).

The work presented here demonstrates that CPT-11 is highly active against three neuroblastoma xenografts as it induced complete regressions and significant tumour growth delays in all models treated with short-term schedules (less than 2 weeks). In addition, the pPNET xenograft model proved to be highly sensitive with 100% of tumour-free survivors. The intermittent schedule was consistently found to be less effective than the daily schedule. CPT-11 is clearly active against neuroblastoma and pPNET xenografts, as its anti-tumour activity was retained at doses lower than the optimal dosage. These results are in good agreement with those of Komuro *et al.* (1994) who showed that CPT-11, administered i.p., induced significant tumour growth inhibition in the TNB9 neuroblastoma xenograft model, although no complete tumour regression was reported. CPT-11 was also found to be active against other paediatric cancer

**Table III** Anti-tumour activity of five anti-cancer drugs against SK-N-MC

| *Xenograft/ drug* | *DT mean ± s.d. (days)* | *Dose (mg kg$^{-1}$ day$^{-1}$)* | *Schedule/ route* | *Total dose (mg kg$^{-1}$)* | n | *BWL (%)* | *Toxic deaths* | *CR* | *PR* | *TGD (days)* | *Tumour-free survivors* |
|---|---|---|---|---|---|---|---|---|---|---|---|
| Cyclophosphamide | 5.4 ± 0.7 | 400 | Single/i.p. | 400 | 10 | 0 | 0 | 9 | 1 | > 107 | 9/10 on day 120 |
| Thiotepa | 6.2 ± 1.6 | 24 | Single/i.p. | 24 | 9 | 0.5 | 1 | 8 | – | > 120 | 6/8 on day 120 |
| Cisplatin | 7.3 ± 2.3 | 5 | day 0, day 4/ i.v. | 10 | 8 | 0 | 0 | 1 | – | 9 | 1/8 on day 122 |
| | | 7 | day 0, day 4/ i.v. | 14 | 8 | 5 | 0 | 1 | 2 | 14 | 1/8 on day 122 |
| | | 10 | day 0, day 4/ i.v. | 20 | 8 | 13 | 1 | 3 | 3 | 25 | 2/7 on day 122 |
| Etoposide | 8.0 ± 1.1 | 17 | Daily × 5/ i.v. | 85 | 8 | 4 | 0 | 0 | 0 | 0 | 0/8 |
| | | 25 | Daily × 5/ i.v. | 125 | 8 | 13 | 2 | 0 | 0 | 0 | 0/6 |
| Taxol | 6.2 ± 3.6 | 12 | Daily × 7/ i.p. | 84 | 5 | 0 | 0 | 0 | 0 | 5 | 0/5 |
| | | 16 | Daily × 7/ i.p. | 112 | 5 | 3 | 0 | 0 | 0 | 16 | 0/5 |
| | | 18 | Daily × 7/ i.p. | 126 | 5 | 14 | 4 | 0 | 0 | NA | |

Doubling time was measured in each control group during the exponential phase of tumour growth; *n*, number of animals per group; BWL, maximal body weight loss; CR, complete tumour regression; PR, partial tumour regression; TGD, tumour growth delay; NA, not available.

**Table IV** Anti-tumour activity of five anti-cancer drugs against IGR-N835

| *Xenoraft/ drug* | *DT mean ± s.d. (days)* | *Dose (mg kg$^{-1}$ day$^{-1}$)* | *Schedule/ route* | *Total dose (mg kg$^{-1}$)* | n | *BWL (%)* | *Toxic deaths* | *CR* | *PR* | *TGD (days)* | *Tumour-free survivors* |
|---|---|---|---|---|---|---|---|---|---|---|---|
| Cyclophosphamide | 3.2 ± 0.9 | 400 | Single/i.p. | 400 | 8 | 0 | 0 | 2 | 2 | 28 | 0/8 |
| Thiotepa | 3.2 ± 0.8 | 24 | Single/i.p. | 24 | 10 | 0 | 0 | 0 | 5 | 13 | 0/10 |
| Cisplatin | 3.5 ± 0.9 | 3.5 | day 0, day 4/ i.v. | 7 | 8 | 3 | 0 | 0 | 0 | 12 | 0/8 |
| | | 5 | day 0, day 4/ i.v. | 10 | 8 | 8 | 0 | 1 | 5 | 19 | 1/8 on day 151 |
| | | 7 | day 0, day 4/ i.v. | 14 | 8 | 12.7 | 0 | 5 | 3 | 21 | 1/8 on day 151 |
| Etoposide | 4.1 ± 0.6 | 17 | Daily × 5/i.v. | 85 | 8 | 5.5 | 0 | 0 | 0 | 4 | 0/8 |
| | | 25 | Daily × 5/i.v. | 125 | 8 | – | 8 | NA | NA | | |
| Taxol | 3.5 ± 1.6 | 12 | Daily × 7/i.p. | 84 | 9 | 0 | 0 | 0 | 0 | 3 | 0/9 |
| | | 16 | Daily × 7/i.p. | 112 | 9 | 8 | 0 | 0 | 0 | 10 | 0/9 |
| | | 20 | Daily × 7/i.p. | 140 | 9 | 10 | 9 | 0 | 0 | NA | – |

Doubling time was measured in each control group during the exponential phase of tumour growth; *n*, number of animals per group; BWL, maximal body weight loss; CR, complete tumour regression; PR, partial tumour regression; TGD, tumour growth delay; NA, not available.

xenografts, including rhabdomyosarcomas (Houghton *et al.*, 1993) and medulloblastomas (Vassal *et al.*, 1994). Houghton showed that CPT-11 active against five of six childhood rhabdomyosarcoma xenograft models, even at dosages below the maximum tolerated dose (MTD) (Houghton *et al.*, 1993). In addition, a recent study suggested that the use of protracted schedules may increase the therapeutic activity of CPT-11 (Houghton *et al.*, 1995).

Previous studies have shown that CPT-11 is active against multidrug-resistant cell lines (Kunimoto *et al.*, 1987; Tsuruo *et al.*, 1988). Houghton also found that CPT-11 retained its activity *in vivo* against a vincristine-resistant rhabdomyosarcoma xenograft (Houghton *et al.*, 1993). This is important as the human *mdr1* gene is overexpressed in neuroblastomas and several clinical studies have noted a relationship between *mdr1* overexpression and a poor prognosis in neuroblastoma patients (Bourhis *et al.*, 1989; Chan *et al.*, 1991). It is noteworthy that our study demonstrates CPT-11 activity against neuroblastoma xenografts overexpressing the human *mdr1* gene.

The activity of five anti-cancer drugs was evaluated against SK-N-MC and IGR-N835 to validate our models in comparison with the established chemosensitivity of pPNETs and neuroblastomas in children. Cyclophosphamide and etoposide are currently used in conventional chemotherapy protocols to treat patients with neuroblastoma and Ewing's sarcoma (Hayes *et al.*, 1989; Méresse *et al.*, 1993; Kushner *et al.*, 1994). Cisplatin is a major drug used to treat neuroblastoma (Philip *et al.*, 1987; Kushner *et al.*, 1994). Thiotepa is an alkylating agent used at a high dose before autologous bone marrow stem cell support in adult and childhood malignancies (Wolff *et al.*, 1990). Taxol, a new anti-cancer agent, is currently being investigated in phase I and II clinical trials in children with solid tumours and leukaemia (Hurwitz *et al.*, 1993; Kretschmar *et al.*, 1995). The two xenografts were clearly sensitive to alkylating agents (cyclophosphamide, thiotepa) and cisplatin, with SK-N-MC being more sensitive than IGR-N835. This is in good agreement with the established sensitivity of pPNETs and neuroblastomas (Hayes *et al.*, 1989; Méresse *et al.*, 1993; Kushner *et al.*, 1994). Conversely, the two xenografts were refractory to taxol and etoposide. A recent up-front phase II study on taxol has shown an 18% response rate in neuroblastoma patients (Kretschmar *et al.*, 1995). Etoposide is widely used in paediatric oncology in combination with other drugs such as cyclophosphamide and platinum compounds (cisplatin or carboplatin) (Philip *et al.*, 1987; Méresse *et al.*, 1993). However, phase II studies of single-agent etoposide showed very low response rates (<10%) in neuroblastoma and Ewing's sarcoma patients (Kung *et al.*, 1988). Thus, the CPT-11 activity was observed in two xenografts, which seems to reflect the sensitivity of neuroblastomas and pPNETs observed in children.

In conclusion, CPT-11 was found to be highly active against pPNET and neuroblastoma xenografts that exhibited the biological features of poor prognosis tumours in children. The high activity of CPT-11 observed in this study compared with that of conventional anti-cancer drugs used to treat children with neuroblastoma and pPNET suggests that CPT-11 deserves prompt evaluation in paediatric oncology.

**Acknowledgements**

We thank Dr Anne Mathieu-Boué and Dr Mondher Majhoubi from Laboratoire Bellon, Neuilly-sur-Seine, for helping us to initiate this study and for providing us with CPT-11; the staff of the Animal Experimentation Unit, Institut Gustave-Roussy, for the care of athymic mice; Dr Gauthier, Hôpital de Bicêtre, Le Kremlin-Bicêtre, and Pr Hélardot, Hôpital Saint-Vincent-de-Paul, Paris, for providing us with patient tumour samples; and Loran Saint-Ange for editing the manuscript. This work was supported by grants from the Association pour la Recherche contre le Cancer, Villejuif, the Fédération Nationale des Centres de Luttre Contre le Cancer (FNCLCC) and the Ligue Contre le Cancer.

### References

ABIGERGES D, CHABOT GG, ARMAND JP, HERAIT P, GOUYETTE A AND GANDIA D. (1995). Phase I and pharmacologic studies of the camptothecin analog irinotecan administered every 3 weeks in cancer patients. *J. Clin. Oncol.*, **13**, 210–221.

BETTAN-RENAUD L, BAYLE C, TEYSSIER JR AND BENARD J. (1989). Stability of phenotypic and genotypic traits during the establishment of a human neuroblastoma cell line, IGR-N-835. *Int. J. Cancer*, **44**, 460–466.

BIEDLER JL, HELSON L AND SPENGLER BA. (1973). Morphology and growth, tumorigenicity and cytogenetics of human neuroblastoma cells in continuous culture. *Cancer Res.*, **33**, 2643–2652.

BISSERY MC, MATHIEU-BOUE A AND LAVELLE F. (1992). Experimental antitumour activity of CPT-11 in vitro and in vivo. *Ann. Oncol.*, **3** (suppl. 1), 82.

BOURHIS J, BENARD J, HARTMANN O, BOCCON-GIBOD L, LEMERLE J AND RIOU G. (1989). Correlation of MDR1 gene expression with chemotherapy in neuroblastomas. *J. Natl Cancer. Inst.*, **81**, 1401–1405.

BOVEN E, WINOGRAD B, GODSTAD O, LOBBEZOO MW AND PINEDO HM. (1988). Preclinical phase II studies in human tumor lines: a european multicenter study. *Eur. J. Cancer Clin. Oncol.*, **24**, 567–573.

BRODEUR GM, SEEGER RC, SCHWAB M, VARMUS HE AND BISHOP JM. (1984). Amplification of N-myc in untreated human neuroblastomas correlates with advanced disease stage. *Science*, **224**, 1121–1124.

CHAN H, HADDAD G, THORNER P, DEBOER G, PING LIN Y, ONDRUSEK N, YEGER H AND LING V. (1991). P-glycoprotein expression as a predictor of the outcome of therapy for neuroblastoma. *N. Engl. J. Med.*, **325**, 1608–1614.

CHEN P, LIN HH AND WEISSMAN BE. (1995). A functional analysis of tumor suppressor activity for peripheral neuroepitheliomas by monochromosome transfer.*Oncogene*, **10**, 577–586.

DELATTRE O, ZUCMAN J, PLOUGASTEL B, DESMAZE C, MELOT T, PETER M, KOVAR H, JOUBERT I, DE JONG P, ROULEAU G, AURIAS A AND THOMAS G. (1992). Gene fusion with an ETS DNA-binding domain caused by chromosome translocation in human tumors. *Nature*, **359**, 162–165.

DUTRILLAUX B AND LEJEUNE J. (1971). On a new technique of human karyotype study. *CR Acad. Sci. Paris*, **272**, 2638–2640.

FERRANDIS E, DA SILVA J, RIOU G AND BENARD J. (1994). Coactivation of the MDR1 and MYCN genes in human neuroblastoma cells during the metastatic process in the nude mouse. *Cancer Res.*, **554**, 2256–2261.

FONG CT, DRACOPOLI NC, WHITE PS, MERRILL T, GRIFFITH RC, HOUSMAN DE AND BRODEUR GM. (1989). Loss of heterozygosity for the short arm of chromosome 1 in human neuroblastomas: correlation with N-myc amplification. *Proc. Natl Acad. Sci. USA*, **86**, 3753–3757.

FRIEDMAN HS, SCHOLD SC AND BIGNER DD. (1986). Chemotherapy of subcutaneous and intracranial human medulloblastoma xenografts in athymic mice. *Cancer Res.*, **46**, 224–228.

GEORGE BA, YANIK G, WELLS RJ, MARTIN LW, SOUKUP S, BALLARD ET, GARTSIDE PS AND LAMPKIN BC. (1993). Growth patterns of human neuroblastoma xenografts and their relationship to treatment outcome. *Cancer*, **73**, 3331–3339.

HAYASHI Y, KANDA H, INABA T, HANADA R, NAGAHARA N, RUCHI H AND YAMAMOTO K. (1989). Cytogenetic findings and prognosis in neuroblastoma with emphasis on marker chromosome 1. *Cancer*, **63**, 126–132.

HAYES FA, THOMPSON EL, MEYER WH, KUN L, PARHAM D, RAO B, KUMAR M, HANCOCK M, PARVEY L, MAGILL L AND HUST O. (1989). Therapy for localized Ewing's sarcoma of bone. *J. Clin. Oncol.*, **7**, 208–213.

HOUGHTON PJ, CHESCHIRE PJ, HALLMAN JC, BISSERY MC, MATHIEU-BOUE A AND HOUGHTON JA. (1993). Therapeutic activity of the topoisomerase I inhibitor 7-ethyl-10-(4[1-piperidino]-1-piperidino)-carbonyloxy-camptothecin against human tumor xenografts: lack of cross resistance in vivo in tumors with acquired resistance to the topoisomerase I inhibitor 9-dimethylaminomethyl-10-hydroxycamptothecin. *Cancer Res.*, **53**, 2823–2829.

HOUGHTON PJ, CHESCHIRE PJ, HALLMAN II JD, LUTZ L, FRIEDMAN HS, DANKS MK AND HOUGHTON JA. (1995). Efficacy of topoisomerase I inhibitors, topotecan and irinotecan, administered at low dose levels in protracted schedules to mice bearing xenografts of human tumors. *Cancer Chemother. Pharmacol.*, **36**, 393–403.

HSIANG YH, LIHOU LF AND LIU LF. (1989). Arrest of DNA replication by drug-stabilized topoisomerase I-DNA cleavable complexes as a mechanism of cell killing by camptothecin. *Cancer Res.*, **49**, 5077–5082.

HURWITZ CA, RELLING MW, WEITMAN SD, RAVINDRANATH Y, VIETTI TJ, STROTHER DR, RAGAD AH AND PRATT CB. (1993). Phase I trial of paclitaxel in children with refractory solid tumors. *J. Clin. Oncol.*, **11**, 2324–2329.

KAWATO Y, FURUTA T, AONUMIA M, YASUOKA M, YOKOKURA T AND MATSUMOTO K. (1991). Antitumor activity of a camptothecin derivative, CPT-11, against human tumor xenografts in nude mice. *Cancer Chemother. Pharmacol.*, **28**, 192–198.

KOMURO H, LI P, TSUCHIDA Y, YOKOMORI K, NAKAJIMA K, AOYAMA T, KANEDO M AND KANEDA N. (1994). Effects of CPT-11 (a unique DNA topoisomerase I inhibitor) on a highly malignant xeno-transplanted neuroblastoma. *Med. Ped. Oncol.*, **23**, 487–492.

KRETSCHMAR C, KLETZEL M, MURRAY K, JOSHI V, SMITH E, PAO R AND CASTLEBERRY R. (1995). Upfront phase II therapy with taxol and topotecan in untreated children (<365 days) with disseminated (INSS stage 4) neuroblastoma. A Pediatric Oncology Group study. *Med. Ped. Oncol.*, **25**, 243.

KUNG F, FAYES A, KRISHNER J, MAHONEY D, LEVENTHAL B, BRODEUR G, BERRY DH, DUBOWY R AND TOLEDANO S. (1988). Clinical trial of etoposide (VP-16) in children with recurrent malignant solid tumors. *Invest. New Drugs*, **6**, 31–36.

KUNIMOTO T, NITTA K, TANAKA T, UEHARA N, BABA H, TAKEUCHI M, YOKOKURA T, SAWADA S, MIYASAKA T AND MUTAI M. (1987). Antitumor activity of 7-ethyl-10-(4[1-piperidino]-1-piperidino)-carbonyloxy-camptothecin, a novel water soluble derivative of camptothecin, against murine tumors. *Cancer Res.*, **47**, 5944–5947.

KUSHNER BH, LAQUAGLIA MP, BONILLA MA, LINDSLEY K, ROSENFIELD N, YEH S, EDDY J, GERALD WL, HELLER G AND CHEUNG NV. (1994). Highly effective induction therapy for stage 4 neuroblastoma in children over 1 year of age. *J. Clin. Oncol.*, **12**, 2607–2613.

MASUDA N, FUKUOKA M, KUSUNOKI Y, MATSUI K, TAKIFUJI N, KUDOH S, NEGORO S, NISHIOKA M, NAKAGAWA K AND TAKADA M. (1992). CPT-11: a new derivative of camptothecin for the treatment of refractory or relapsed small-cell lung cancer. *J. Clin. Oncol.*, **10**, 1225–1229.

MÉRESSE V, VASSAL G, MICHON J, DE CERVENS C, COURBON B, RUBIE H, PEREL Y, LANDMAN J, CHASTAGNIER P, DE VALCK C AND HARTMANN O. (1993). Combined continuous infusion etoposide with high-dose cyclophosphamide for refractory neuroblastoma: a phase II from the Société Française d'Oncologie Pédiatrique. *J. Clin. Oncol.*, **11**, 630–637.

PHILIP T, GHALIE R, PINKERTON R, ZUCKER JM, BERNARD JL, LEVERGER G AND HARTMANN O. (1987). A phase II of high-dose cisplatin and VP-16 in neuroblastoma: a report from the Société Française d'Oncologie Pédiatrique. *J. Clin. Oncol.*, **5**, 941–950.

POMMIER Y, TANIZAWA A AND KOHN KW. (1994). Mechanisms of topoisomerase I inhibition by anticancer drugs. In: *Advances in Pharmacology*, 29B, Liu LF (ed.), pp. 73–92. Academic Press Inc: San Diego.

POTMESIL M. (1994). Camptothecins: from bench research to hospital wards. *Cancer Res.*, **54**, 1431–1439.

TANIZAWA A, FUJIMORI A, FUJIMORI Y AND POMMIER Y. (1994). Comparison of topoisomerase I inhibition, DNA damage, and cytotoxicity of camptothecin derivatives presently in clinical trials. *J. Natl Cancer Inst.*, **86**, 836–842.

TSURUO T, MATSUZAKI T, MATSUSHITA M, SAITO H AND YOKOKURA T. (1988). Antitumor effect of CPT-11, a new derivative of camptothecin, against pleiotropic drug resistant tumors in vitro and in vivo. *Cancer Chemother. Pharmacol.*, **21**, 71–74.

VASSAL G, MORIZET J, BISSERY MC, BOLAND I, ARDOUIN P, MATHIEU-BOUE A AND GOUYETTE A. (1994). Activity of the camptothecin analog CPT-11 (irinotecan) against medulloblastoma xenografts. *Proc. Am. Assoc. Cancer Res.*, **35**, 366.

WINOGRAD B, LOBBEZOO MW AND PINEDO HM. (1988). Proposal for the application of xenografts in screening for new anticancer agents and in selecting tumor types for phase II clinical trials. In *Human Tumor Xenografts in Anticancer Drug Development.* Winograd B, Peckham MJ, Pinedo HM (eds), pp. 111–114. Springer: Berlin.

WOLFF SN, HERZIG RH, FAY JW, LEMAISTRE CF, BROWN RA, FREI-LAKR D, STRANJORD S, GIANNONE L, COCCIA P, WEICK JL, ROTHMAN SA, KRUPP KR, LOWDER J, BOLWELL B AND HERZIG GP. (1990). High-dose *N,N'N''*-triethylenethiophosphoramide (thiotepa) with autologous bone marrow transplantation: phase I studies. *Semin. Oncol.*, **17** (Suppl. 3), 2–6.